# Study Controls: Ashby Responds

**Published:** 2005-09

**Authors:** John Ashby

**Affiliations:** Syngenta Central Toxicology Laboratory Alderley Park, Cheshire, United Kingdom E-mail: john.ashby@syngenta.com

Figure 1 is the relevant summary figure (Figure 8) from our article (Ashby et al. 2004). Our point in the article, as well as now, is that it is incumbent upon each investigator to accept, to study, and where possible, to understand the extent, nature, and origins of variability (within and between experiments) of the critical assay parameter. If you do not know why the assay parameter varies naturally with time, or between experiments, it becomes difficult to interpret small perturbations of the parameter induced in a chemical toxicity study. This was the problem we faced when we tried to explain our inability, over four extensive studies (Ashby et al. 2003), to confirm the effects that Sakaue et al. (2001) reported for bisphenol A (BPA). The control values for daily sperm production (DSP) in Sprague-Dawley rats over our four experiments (Figure 1) varied little, despite the use of three different rodent diets and a variety of physical test conditions (changes in bedding and caging). We also noted (Ashby et al. 2004) that Sakaue et al. reported similar control DSP values for Holtzman rats (Sakaue et al. 1999) and Sprague-Dawley rats (Sakaue et al. 2001; Figure 1). The most interesting aspect of the data in Figure 1 is the extent of variability in control DSP values reported by Sakaue et al. (2001) for their two experiments on BPA in Sprague-Dawley rats. It is important to understand the origins of these variations in control DSP values between similar experiments before interpreting small chemically induced perturbations in DSP values with confidence. Equally, by paying attention to the origins of control variability, we were able to show that two chemicals we had previously considered to be negative in the rodent uterotrophic assay were, in fact, weakly positive (Ashby et al. 2004). Stable control values for an assay lead to the generation of sound assay data.

## Figures and Tables

**Figure 1:**
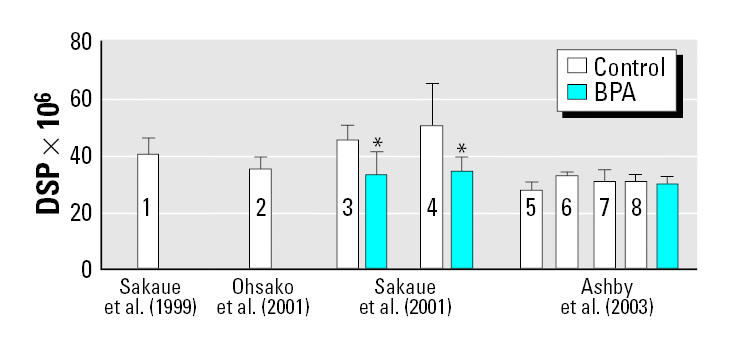
Comparison of control DSP (mean ± SD) reported from the same laboratory [Ohsako et al. (2001) and Sakaue et al. (1999, 2001)] and a different laboratory (Ashby et al. 2003) with the greatest effect induced by BPA (Sakaue et al. 2001). A range of BPA doses was used in these experiments, and only the dose that induced the greatest effect in each experiment is shown: 20 μg/kg (Sakaue et al. 1999); 200 μg/kg (Sakaue et al. 2001); 200 mg/kg (Ashby et al. 2003). The effect of BPA is not significantly different from the control reported by Ohsako et al. (2001; bar 2: one- or two-sided Student's *t*-test). Sakaue et al. (1999) and Ohsako et al. (2001) used Holtzman rats, and Sakaue et al. (2001) and Ashby et al. (2003) used Sprague-Dawley rats. However, the identical control DSP values for Holtzman rats (Sakaue et al. 1999, bar 1) and Sprague-Dawley rats (Sakaue et al. 2001; bar 3) indicate that rat strain is not a key variable on control DSP values and that, consequently, it is possible to compare data across strains and experiments for that laboratory. Reprinted from Ashby et al. (2004) with permission from *Environmental Health Perspectives*. *Reported by Sakaue et al. (2001) as statistically different from the concurrent control (bars 3 and 4).
